# Human GPR42 is a transcribed multisite variant that exhibits copy number polymorphism and is functional when heterologously expressed

**DOI:** 10.1038/srep12880

**Published:** 2015-08-11

**Authors:** Henry L. Puhl III, Yu-Jin Won, Van B. Lu, Stephen R. Ikeda

**Affiliations:** 1 Section on Transmitter Signaling, Laboratory of Molecular Physiology, National Institute on Alcohol Abuse and Alcoholism, National Institutes of Health, Bethesda, Maryland 20892-9411, USA

## Abstract

FFAR3 (GPR41) is a G-protein coupled receptor for which short-chain fatty acids serve as endogenous ligands. The receptor is found on gut enteroendocrine L-cells, pancreatic β-cells, and sympathetic neurons, and is implicated in obesity, diabetes, allergic airway disease, and altered immune function. In primates, FFAR3 is segmentally duplicated resulting in GPR42, a gene currently classified as a suspected pseudogene. In this study, we sequenced FFAR3 and GPR42 open reading frames from 56 individuals and found an unexpectedly high frequency of polymorphisms contributing to several complex haplotypes. We also identified a frequent (18.8%) structural variation that results in GPR42 copy number polymorphism. Finally, sequencing revealed that 50.6% of GPR42 haplotypes differed from FFAR3 by only a single non-synonymous substitution and that the GPR42 reference sequence matched only 4.4% of the alleles. Sequencing of cDNA from human sympathetic ganglia and colon revealed processed transcripts matching the GPR42 genotype. Expression of several GPR42 haplotypes in rat sympathetic neurons revealed diverse pharmacological phenotypes that differed in potency and efficacy. Our data suggest that GPR42 be reclassified as a functioning gene and that recognition of sequence and copy number polymorphism of the FFAR3/GPR42 complex be considered during genetic and pharmacological investigation of these receptors.

Free fatty acid receptors (FFAR) are a family of seven trans-membrane spanning G-protein coupled receptors (GPCR) that utilize free fatty acids as endogenous agonists. FFAR2 and FFAR3 utilize short-chain fatty acids (SCFA) with 2–4 carbons (acetate, C2; propionate, C3; butyrate, C4) as ligands while FFA1 and FFA4 require longer chain fatty acids (C8–C22) for activation^1^. FFAR2 and FFAR3, originally termed GPR43 and GPR41, respectively, were de-orphanized in 2003 by two groups working independently^2^,^3^. Both FFAR2 and FFAR3 are heavily expressed in enteroendocrine cells (primarily I- and L-cells) of the distal small intestine and proximal large intestine where bacterial fermentation of soluble fiber provides a source of SCFAs at concentrations sufficient to activate these relatively low affinity receptors. Recently, the composition of the gut microbial community, or microbiota, have been shown to influence metabolic physiology at least partially via mechanisms dependent on FFAR2^4^ and FFAR3^5^,^6^,^7^. FFAR3 is also implicated in allergic airway disease^8^, diabetes^9^, and immune function^10^. At sites remote from the gut lumen and associated vasculature (e.g., the hepatic portal vein), plasma concentrations of SCFAs are maintained at lower levels in non-ruminants and thus mechanisms of endogenous receptor activation are less clear. For example, FFAR3 is expressed in postganglionic sympathetic neurons distant from the gut lumen and the ketone body, β-hydroxybutyrate (BHB), has been put forth as a potential endogenous ligand^11^,^12^,^13^. In addition, oxidative metabolism of ethanol in humans^14^,^15^ results in a pharmacological source of plasma C2 at concentrations potentially relevant to FFAR2/FFAR3 receptor activation.

Extrapolating the role of FFAR3 from rodents to humans is complicated by two factors. First, mouse FFAR3, which is about 80% similar to human FFAR3, displays distinctive pharmacological profiles^16^. Second, a tandem segmental duplication of the genomic region containing the open reading frame (ORF) of FFAR3^17^ is present in primates but not other mammals. The duplicated FFAR3 ORF, termed GPR42 in humans, was originally declared a pseudogene based on a lack of functional activity when the GPR42 ORF (Genbank: NG_008348), which is contained within a single exon, was heterologously expressed in various yeast and mammalian systems^2^. The predicted protein product for GPR42 varied by six amino acids from FFAR3 with the R174W substitution determined as critical for ablating GPR42 function. The designation of GPR42 as a nonfunctioning pseudogene and the lack of an equivalent duplication in rodents suppressed further study. However, a detailed study of GPR42 ORF variation using restriction enzyme polymorphism analysis of amplified genomic regions demonstrated that the R174W substitution occurred in only 39% of GPR42 ORFs^18^. Moreover, sequencing of selected FFAR3 and GPR42 ORFs showed variations at 5 of 6 positions differing from the Genbank reference sequences. This work thus raised the question of whether the GPR42 ORF was a pseudogene and pointed out the potential uncertainty in determining FFAR3 or GPR42 genomic sequences due to the high degree of sequence identity between the two regions harboring the ORFs. It was also emphasized that standard techniques used for determining mRNA expression, including Northern blot analysis, TaqMan, and RT-PCR would be unlikely to discriminate between transcripts arising from FFAR3 and GPR42^18^.

In this study, we wished to determine whether GPR42 is properly classified as a pseudogene or should be reclassified as a functional gene. To this end, we examined three different aspects of GPR42 gene structure and function. First, we extended the work of Liaw and Connolly^18^ by amplifying specific genomic regions containing FFAR3 and GPR42 ORFs from fifty six individuals, cloning individual alleles, and sequencing the ORFs for FFAR3 and GPR42. Our results indicate that both FFAR3 and GPR42 are highly polymorphic with numerous positions yielding minor allele frequencies greater than 30%. Moreover, the majority of GPR42 ORFs lacked the R174W substitution and were thus predicted to be functional. None of the ORFs examined contained frame-shifting indels or premature stop codons. Second, we amplified mRNA from human sympathetic ganglia and colon and identified cDNA sequences that originated from GPR42 alleles. Finally, we heterologously expressed several of the human GPR42 ORFs in rat sympathetic neurons and demonstrated functional coupling to N-type Ca^2+^ channels following application of agonist. Taken together, these results provide evidence that human GPR42 is both transcribed and potentially produces a functional protein. Thus, the status of GPR42 should be changed from pseudogene to functional and the gene product(s) of GPR42 assigned to a member of the FFA receptor family.

## Results

**Figure 1:**
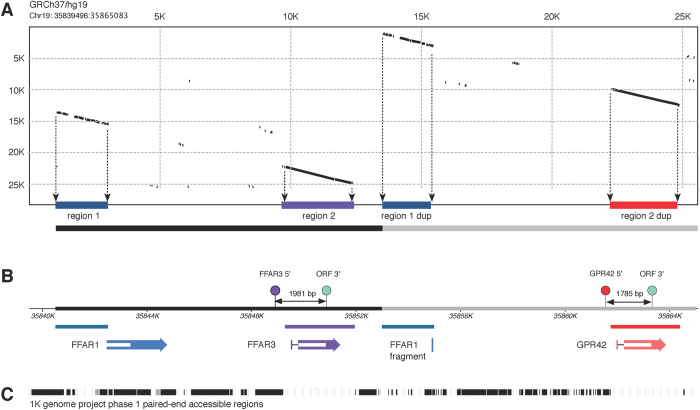
Tandem segmental duplication of FFAR1 and FFAR3. (**A**) Dot plot of a 25.6 kb region from human chromosome 19. The plot was generated with a Pustell DNA Matrix (scoring matrix DNA identity, window size of 30, similarity 90%) in MacVector version 13.5. Diagonals represent regions of sequence identity (the self identity diagonal was removed for clarity) and projected to the abscissa (colored bars). The black and gray bars (bottom) represent the presumed original 12.5 kb duplication. (**B**) Annotation of genes and primers. Colored arrows represent genes with the white bar depicting the open reading frame. FFAR1, FFAR3 and GPR42 are shown in blue, purple, and red, respectively. The small portion of FFAR1 retained in the duplicated region is labeled as FFAR1 fragment (blue bar). Location of primers used for PCR amplification of FFAR3 and GPR42 ORFs are shown as balls above the sequence map along with the predicted amplicon length. (**C**) Composite accessible region mask from 1000 genomes phase 1. The filled area represents regions accessible to accurate analysis with short read sequencing.

The human gene for FFAR3 resides at chromosomal locus 19q13.1 between FFAR1 (GPR40) and FFAR2 (GPR43). This syntenic relationship appears preserved in most mammals suggesting that the gene family arose from an ancient gene duplication event. Starting with primates, however, a tandem segmental duplication of a 12.5 kb piece encompassing both FFAR1 and FFAR3 is present. A Pustell DNA matrix dot plot (Fig. 1A) of human chromosome 19 region 35839496:35865083 (GRCh37/hg19) illustrates the duplicated segments (black and gray bar; bottom) and, within these segments, two smaller regions (denoted region 1 and 2 in Fig. 1A) have retained a high degree of sequence identity (>90%). Region 1 (dark blue bar) resides primarily upstream to the open reading frame (ORF) of FFAR1 and extends about 46 bases into the ORF (Fig. 1B;^18^). Hence, the Region 1 duplicon (region 1 dup) may retain FFAR1 promoter elements but is not predicted to code for a functional protein. The function of this region and the evolutionary pressure maintained to conserve sequence identity is unknown. Conversely, region 2 (purple bar) encompasses both exon 1 and 2 of FFAR3. Therefore, the region 2 duplicon (red bar), which retains extremely high sequence identity (≈98%) for about 2.5 kb, includes the entire ORF and 3′-UTR containing exon, an upstream exon encoding most of the 5′-UTR, and an additional 500 bp of the putative upstream intragenic region that may contain a promoter region (Fig. 1B, red elements). The ORF in the region 2 duplicon is sometimes denoted GPR42p (we will use GPR42 hereafter) based on the lack of functional activity of the heterologously expressed GPR42 reference sequence (Genbank: NG_008348) in cellular assays^2^. However, the seminal work by Liaw and Connolly^18^ provide evidence that the reference sequence for GPR42 may not be representative of the population examined (not specified) in that study. Because of the very high sequence identity in FFAR3/GPR42 regions, most short read high-throughput technologies are unable to reliably assign sequences to these regions as illustrated in the accessible region mask (1 K genomes project, phase 1) depicted in Fig. 1C. Therefore, little is known about the sequence variability of either the FFAR3 or GPR42 ORF on a population basis.

To address this question, we adapted the methodology of Liaw and Connolly^18^ to sequence the FFAR3 and GPR42 ORFs from the genomic DNA of 56 individuals. Briefly, PCR amplicons of the FFAR3 (1.98 kb) and GPR42 (1.78 kb) ORF were generated with a high fidelity polymerase from primers targeted to unique regions upstream of the respective ORFs (Fig. 1B, purple and red balls) plus a common downstream primer (Fig. 1B, cyan ball). PCR products were subcloned and DNA from 5–8 individual colonies sequenced to determine allelic variation.

### Sequence of the FFAR3 open reading frame

Initial results from FFAR3 and GPR42 sequencing revealed an unexpected degree of variation so nucleotides positions within the ORF that varied from the FFAR3 reference sequence (Genbank: NM_005304) were assigned an index value to facilitate sorting. A table listing the assigned index position, nucleotide position (relative to the A of the start codon), residue number with codon position, and amino acid change, if any, are show in figure S1A. The affected residues shown in a GPCR snake diagram are illustrated in figure S1B. All affected positions were bi-allelic.

Figure S2A depicts the sequencing results for FFAR3 from 56 individuals. The y-axis represents the SNP index position described in Fig. S1A. The x-axis represents alleles with adjacent columns representing the same individual. The first 50 individuals were sequenced from an existing gDNA sample cohort (B01–B50). The next 5 individuals (alleles 100–109) were sequenced from gDNA isolated from sympathetic ganglia obtained from the NDRI for which cDNA was also sequenced (CG01–05). The final sample was obtained from an NDRI human colon sample (C01). Each filled block represents a deviation from the FFAR3 reference sequence. To visualize individual isoforms, each column (allele) was converted to a 23-bit unsigned integer based on a position index (a variation from the reference sequence considered as a 1) and sorted with the most frequent isoforms to the left. Isoform nomenclature was based on frequency and grouping with synonymous isoforms receiving a sub-designation.

**Figure 2:**
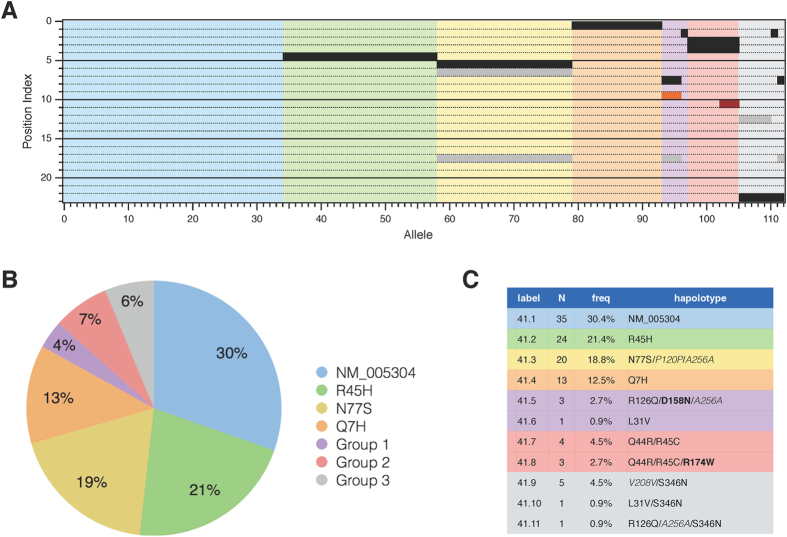
Sequence of FFAR3 alleles. (**A**) Raster diagram of FFAR3 ORF sequences from a cohort of 56 individuals. Each row represents a SNP index position as defined in Fig. S1A. A column represents one of the alleles sequenced from each individual. A filled box represents an alteration from the FFAR3 reference sequence (Genbank: NM_005304). Black and gray fills represent non-synonymous and synonymous polymorphisms, respectively. Orange fill (D158N) represents a polymorphism that affects constitutive receptor activity. Red fill (R174W) represents a polymorphism associated with decreased receptor activity. The columns have been sorted by haplotype (the unsorted columns are shown in Fig. S2A). Pastel colors correspond to groupings shown in panels B and C. (**B**) Pie chart showing fractional representation of the major haplotypes and groups of related haplotypes. (**C**) Table of FFAR3 variants.

From this analysis (Fig. 2B,C), we see that the reference protein sequence (denoted 41.1; Genbank: NP_005925) represented about 30% of the alleles. Isoforms 41.2–41.4 comprise an additional 53% of the sequences with each possessing a single non-synonymous mutation and, for 41.3, two additional synonymous variations. The remaining variants were manually grouped based on sequence characteristics. Group 1 consists of variants with polymorphisms not found in GPR42. The main variant in this group, 41.5, contained the D158N substitution (Fig. 2A, orange bar) that mutagenesis studies indicate results in increased constitutive activity^19^. Group 2 consists of variants with alterations at positions that were common in GPR42. The 41.8 variant contains the R174W substitution (Fig. 2A, red bar) that is predicted from earlier studies to severely decrease efficacy^2^. Finally, group 3 isoforms all possess the S346N variation that was present in every GPR42 allele we sequenced (see below). The 41.9 variant has the same predicted protein sequence as the most common GPR42 variant although it contains an additional synonymous polymorphism not present in GPR42 (see below). These data show an increased frequency of genetic variability in the FFAR3 with several non-synonymous variations predicted to affect receptor function.

### A structural variation produces copy number polymorphism in GPR42

**Figure 3:**
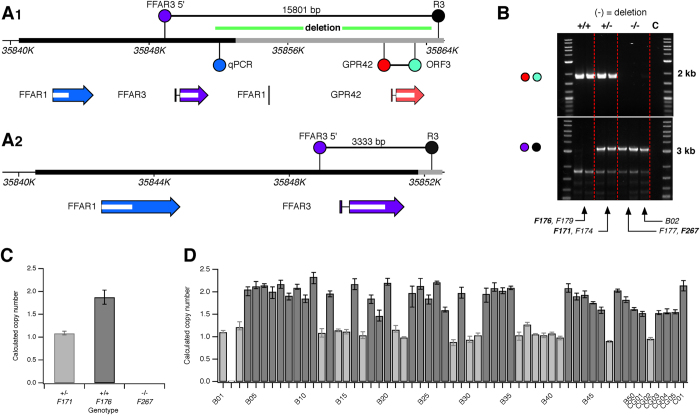
GPR42 copy number polymorphism resulting from a 12.5 kb deletion. (**A1**) Gene map as shown in Fig. 1B with the deletion structural variation (dbVar: esv2678346) shown as a green bar. A PCR primer set used to detect the deletion is shown (purple and black balls). The predicted amplicon of 15.8 kb was not produced with the experimental conditions employed when GPR42 was present. TaqMan® copy number qPCR primer location is indicated with the blue ball. (**A2**) Gene map following deletion of the 12.5 kb sequence. The exact boundaries of the deletion are assumed to be a homologous sequence within region 2 and the region 2 duplicon (Fig. 1A,B). An amplicon of 3.3 kb was produced when the deletion was present. (**B**) Exemplar agarose gel showing PCR amplicons produced with the GPR42 primer set (red and green primers; top gel) and the deletion detection primer set (purple and black primers; bottom gel). A non-specific product was present in the bottom gel below the 2 kb maker. Lanes 1–6 were amplified from gDNA from 1000 genomes Finnish cohort samples (ID below gel). Note that the 1000 genomes identifier format, HG00###, was altered to F### for brevity. The deletion genotype determined from read depth is shown above the gel image. B02 was the sole sample from our cohort for which the GPR42 ORF could not be amplified. (**C**) Validation of TaqMan® copy number qPCR assay using Finnish samples. Each sample was repeated 4 times. Mean copy number (error bars represent sample range) was calculated with ABI CopyCaller™ software. (**D**) Deletion copy number for 56 gDNA samples used in this study. The HDRI colon sample is denoted C01. Light and dark gray bars indicate a copy number of one and two, respectively, as determined by the software.

During the course of this study, we began to suspect a variation in the copy number of GPR42 based on the following lines of evidence. First, in the original description of the human GPR40–43^17^, a cosmid clone (Genbank: U62631) was identified that was missing GPR42 but contained downstream flanking sequence. The authors therefore suggested that GPR42 might represent a polymorphic insert. Second, Liaw and Connolly^18^ analyzed 206 vs. 204 alleles for FFAR3 and GPR42, respectively, although offered no explanation for the mismatch. Similarly, in the course of this study we encountered a single genomic sample, denoted B02, from which we were unable to amplify GPR42 despite repeated attempts with different primer sets. Finally, examination of 1000 Genomes data^20^ with the UCSC Genome Browser^21^ for the region between FFAR3 and GPR42 revealed an approximately 12.5 kb deletion (dbVar: esv2678346; Fig. 3A1) based on read depth analysis that was present in 18.5% of the Phase 1 data. A more detailed examination of these data reveals a large variation in the deletion frequency among different ethnic groups (Fig. S3). For example, the deletion was completely absent in two African populations (LWK and YRI) while in the British cohort (GBR), 11.4 and 40.9% were homozygous and heterozygous, respectively, for the deletion. Despite this evidence, the presence of the deletion has not been directly validated to our knowledge. Accordingly, we examined whether the predicted deletion was present in the cohort under study.

A PCR-based test using two primer sets is shown in Figs 3A1-A2. One primer set (red and cyan balls) was designed to generate a ≈1.8 kb amplicon if GPR42 was present. A second set (purples and black) flanked both FFAR3 and GPR42 and was predicted to generate a ≈3.3 kb amplicon when the deletion was present (Fig. 3A2). Note that the exact boundaries of the deletion were unknown but, assuming the deletion occurred via non-allelic homologous recombination (NAHR), should fall somewhere within the region 2/region 2 duplicon (Fig. 1A). DNA samples from a Finnish cohort (FIN) used in the 1000 Genomes Phase 1 project were obtained and samples containing no deletion (+/+), a single deletion (+/−), or both deletions (−/−) were used to validate the PCR-based test. As predicted, the +/+ samples displayed only the 1.8 kb band, the +/− samples both 1.8 and 3.3 kb bands, and the −/− samples only the 3.3 kb band (Fig. 3B). Note that the sole sample from our cohort, B02, that failed to produce a GPR42 amplicon appears to be homozygous for the deletion. Sequencing the 3.3 kb PCR fragment confirmed the deletion model shown in Fig. 3A2.

An alternative, higher throughput, assay for the deletion based on the TaqMan™ Copy Number Assay (ABI) was run on three of the samples examined in Fig. 3B. The location of the qPCR primer/probe set (Chr.19:35852061) is shown in Fig. 3A1 (blue ball). The probe site lies outside of the sequence homology domain and thus should be absent if the deletion arose from NAHR. The calculated copy number (based on CopyCaller™ v2.0 software, ABI) from the qPCR reaction (Fig. 3C) agreed with the PCR assay (Fig. 3B) and 1000 Genomes data. Thus the assay was run for the 56 samples in our cohort as illustrated in Fig. 3D. The results indicated that one individual, B02, was homozygous for the deletion and thus a predicted knockout of GPR42. Nineteen individuals were heterozygous for the deletion (light gray bars) and thus hemizygotic for GPR42. In summary, 21/112 alleles (18.8%) were positive for the deletion; a result similar to the overall 1000 Genomes Phase 1 data (18.5%).

### Sequence of the GPR42 open reading frame

Sequencing of GPR42 was carried out with the same techniques used to sequence FFAR3. The majority of the sequencing was completed prior to discovering the deletion. Except for one clone, all individuals identified with the copy number assay as GPR42 hemizygotes were previously assigned to be homozygous (i.e., all clones tested had identical sequences). In figure S2B, we denote the deleted allele as a yellow column. Because the sequences were not phased, the deleted allele was arbitrarily assigned to the second column for each individual. The sequencing data shown in figure S2B are presented in comparison with the FFAR3 reference sequence (Genbank: NM_005304) as in Fig. S2A and show a high degree of variation.

**Figure 4:**
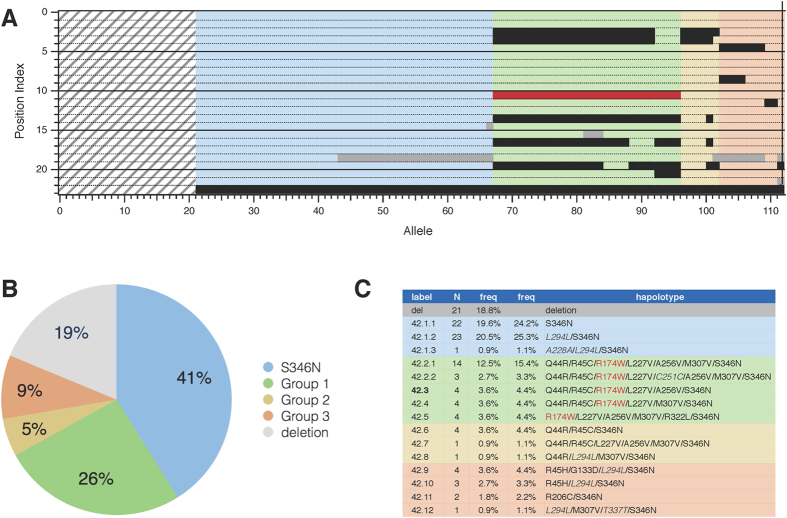
Sequence of GPR42 alleles. (**A**) Raster diagram of GPR42 ORF sequences from a cohort of 56 individuals. Row and columns are as in Fig. 2A. The striped gray group represents deleted alleles as determined in Fig. 3. Pastel colors correspond to groupings shown in panels B and C. (**B**) Pie chart showing fractional representation of the major haplotypes and groups of related haplotypes. (**C**) Table of GPR42 variants. The first frequency column was calculated including the deletion (total = 112). The second frequency column was calculated from sequenced variants (total = 91).

The individual GPR42 haplotypes became more apparent after sorting the data (Fig. 4A) as done earlier (Fig. 2A). The deletion, which affected the copy number of GPR42, is depicted as gray stripes and comprised 18.8% of the potential alleles. Of the GPR42 alleles sequenced, 50.5% (haplotypes 42.1.1–42.1.3; Fig. 4C, second frequency column) predict a translation product that differed from the FFAR3 reference sequence by a single alteration in the last amino acid (S346N). The next block of haplotypes, denoted Group 1 (Fig. 4, green), constitute 31.9% of the sequenced alleles. All Group 1 variants contained the R174W variation (red bar in Fig. 4A) implicated in decreased function and consequent assignment of GPR42 to pseudogene status. Most haplotypes in this group were similar to the GPR42 references sequence (Genbank: AF024689) which consists of six non-synonymous polymorphisms (Q44R/R45C/R174W/L227V/A256V/S346N). It should be noted that the reference sequence, denoted here as haplotype 42.3, comprises just 4.4% of the alleles sequenced. The Group 2 haplotypes (6.6%; yellow) contained some elements found in Group 1 but did not contain the R174W variation. The remaining haplotypes (11%) were assigned to Group 3 (orange) and contained unique variations not found in the FFAR3 haplotypes. All of the GPR42 haplotypes sequenced contained the S346N variation.

These data indicate that the GPR42 ORF sequence is highly heterogeneous within this population and that very few alleles matched the current reference sequence for this gene. Moreover, the vast majority of the haplotypes did not possess the R174W variation that was predicted to produce functional impairment and thus assignment of GPR42 to pseudogene status.

### GPR42 transcript in sympathetic neurons and colon

Although the sequence of GPR42 ORF was highly variable, we did not encounter overt deleterious variations such as frame shift deletions/insertions or premature stop codons. In the cohort sequenced, 50.6% of GPR42 ORFs predicted a protein product that varied from FFAR3 by only a single amino acid in the terminal position thus undermining previous reasoning for assigning GPR42 to pseudogene status. We thus sought to determine whether mRNA for GPR42 was transcribed.

**Figure 5:**
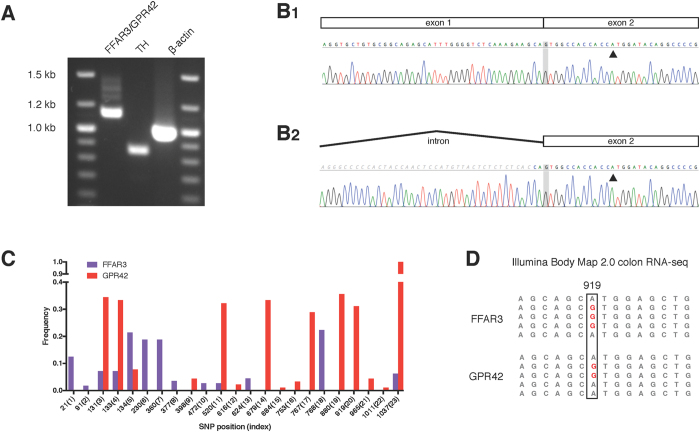
GPR42 transcription. (**A**) Exemplar agarose gel depicting RT-PCR products amplified from human celiac-superior mesenteric ganglia. FFAR3/GPR42: 1.13 kb amplicon that spans the intron between exon 1 and 2. TH: 859 bp amplicon for tyrosine hydroxylase, a marker specific for catecholaminergic neurons. β-actin: 981 bp amplicon for β-actin. (**B1**) Example of sequencing electropherogram for FFAR3/GPR42 cDNA clone showing the splice junction between exon 1 and 2. (**B2**) Sequencing electropherogram for FFAR3/GPR42 clone in which the intron between exon 1 and 2 was retained. Sequences with the intron were not included in analyses. The black triangle represents the first position of the start codon. (**C**) SNP frequency for each index position. FFAR3 and GPR42 SNP frequencies were calculated for 112 and 91 alleles, respectively. The latter number accounts for the GPR42 deletions. SNP position is for nucleotide position within the ORF. Corresponding amino acid positions are shown in Fig. S1A. (**D**) Exemplar sequences from the Illumina Body Map 2.0 colon RNA-seq data set (http://www.ensembl.info/blog/2011/05/24/human-bodymap-2-0-data-from-il) showing several A919G reads (red; 5/18 transcripts assigned to this region were A919G). In our data, A919G was frequently found in GPR42 (30.8%) but never found in FFAR3.

Two factors made detection of GPR42 message challenging. First, the lack of GPR42 in rodents necessitated using human tissue where the distribution of FFAR3 is incompletely characterized. We choose abdominal sympathetic ganglia^11^ and colon^22^ as potential sources of GPR42 message assuming similarity with the FFAR3 expression pattern. Second, because of the high sequence similarity between FFAR3 and GPR42, standard methods of determining mRNA (e.g., Northern blots, TaqMAN qPCR, RT-PCR) would not discriminate between the two gene products^18^. The approach we used was to couple gDNA sequencing of FFAR3/GPR42 alleles, as performed above, with mRNA isolation, reverse transcription, and sequencing of multiple cDNA clones. This allowed unambiguous matching of cDNA sequences to gDNA alleles. Amplicons from RT-PCR of cDNA isolated from human celiac-superior mesenteric ganglia samples are shown in Fig. 5A. Due to the high degree of sequence identity, the primers used were predicted to generate a 1.13 kb amplicon for both FFAR3 and GPR42 (Fig. 5A; lane 1). An 859 bp amplicon for tyrosine hydroxylase (Fig. 5A, lane 2), a marker specific for catecholaminergic neurons, and a 981 bp amplicon (Fig. 5A, lane 3) for β-actin, a general marker, were examined to assess tissue identification and RNA quality. Primers for FFAR3/GPR42 spanned a 230 bp intron that allowed unambiguous identification of sequence originating from processed transcript (Fig. 5B1) versus gDNA or unprocessed transcript (Fig. 5B2). Sequences of cDNA containing the intron were excluded from analysis.

The sequences for gDNA isolated from the celiac-superior mesenteric ganglia are shown in Fig. S2 (alleles 100–110) and the copy number for GPR42 in Fig. 3D (CG01–05). RNA of sufficient quality for subsequent analysis was isolated from CG02–05. The sequencing results for cDNA clones were variable. In one sample, CG05 (0/36), no evidence for GPR42 was found. Conversely, in CG02 (1/30), CG03 (1/30), and CG04 (12/60), sequencing of cDNA clones were in full agreements with at least one of the gDNA GPR42 alleles. We also attempted a similar experiment with pre-existing human colon samples (obtained from NDRI). Of three colon samples, RNA of sufficient quality was isolated from just one. However, in this sample, 16/34 cDNA clones were consistent with a GPR42 allele. Taken together, these data suggest that GPR42 is transcribed in both sympathetic neurons and colon.

In addition to direct sequencing from human tissue, we sought additional evidence for transcription of GPR42 in RNA-Seq databases. As mentioned earlier, most short read next generation sequencing (NGS) techniques have difficulty assigning reads for segmentally duplicated genes with long stretches of nearly identical sequence^23^. However, several variant positions were relatively common amongst GPR42 haplotypes in our cohort (e.g., 679(14), 880(19), and 919(20)) but never present in FFAR3 (Fig. 5C). Another potential marker was the G1037A (S346S) variation which was present in all GPR42 sequences but only rarely (6.4%) seen in FFAR3. Using this information, we found that A919G, a variant found in 30.8% (28/91) of GPR42 alleles but 0% (0/112) of FFAR3 alleles, was present in the Illumina Body Map 2.0 colon RNA-seq study (Fig. 5D). Note that sequences were assigned to both FFAR3 and GPR42 suggesting errors in assignment due to the high degree of sequence identity.

### Heterologous expression and function of GPR42 in rat sympathetic neurons

**Figure 6:**
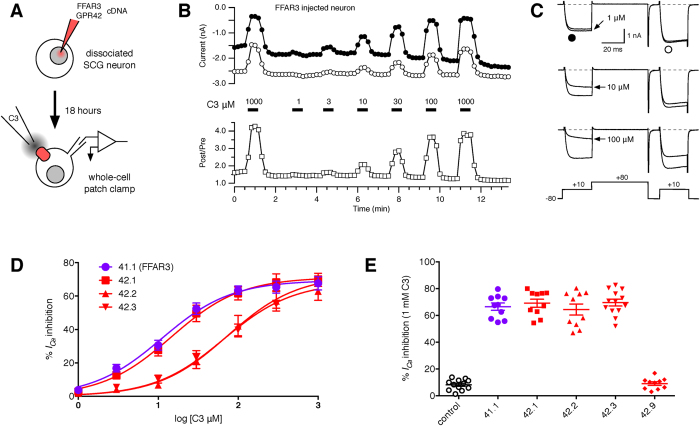
Function of GPR42 haplotype variants. (**A**) Schematic of the electrophysiological assay. FFAR3/GPR42 were expressed in isolated rat superior cervical ganglion (SCG) neurons by intranuclear injection of mammalian expression vectors containing the ORF. After about 18 hours, whole-cell patch-clamp recordings in solutions designed to isolate Ca^2+^ currents were performed. Multiple concentrations of propionate (C3) were applied to the neuron via a multi-bore perfusion device. Activation of FFAR3/GPR42 produced an inhibition of the Ca^2+^ current. (**B,C**) Exemplar time course and current recording traces of a concentration-response protocol. Ca^2+^ currents were evoked with the voltage protocol shown in C (bottom) at 0.1 Hz. Prepulse (filled circles, first epoch to +10 mV) and postpulse (open circles, second epoch to +10 mV) Ca^2+^ current amplitudes were plotted versus time (**B**, top). Application of C3 at the indicated concentration is shown as solid bars (**B**, middle). The postpulse to prepulse amplitude ratio (**B**, bottom) is a parameter related to voltage-dependent modulation; an indicator of Gβγ inhibition. Superimposed current traces for 1, 10, and 100 μM C3 application are shown in **C**. The larger current was acquired just prior to the C3 application. (**D**) Concentration response curves for FFAR3 (41.1; purple filled squares) and GPR42 haplotypes (red filled symbols; 42.1 squares, 42.2 triangles, 42.3 inverted triangles). Mean (±SEM; N varies from 4–13) prepulse Ca^2+^ current inhibition (%) versus log [C3 μM]. Solid lines represent non-linear regression fits to a single site binding isotherm (see Methods). (**E)**. Category scatter plot (efficacy) for Ca^2+^ current inhibition during 1 mM C3 application). Mean (±SEM) Ca^2+^ current inhibition are superimposed on individual determinations. Control neurons were not injected with cDNA. Each category represents a different FFAR3/GPR42 haplotype as labeled.

Finally, we examined whether several of the GPR42 haplotypes were functional when heterologously expressed. The model system (Fig. 6A) used was comprised of sympathetic neurons isolated from rat superior cervical ganglion intranuclearly injected with cDNA encoding an FFAR3 or GPR42 ORF. Following overnight incubation, receptor efficacy and potency were evaluated using inhibition of Ca^2+^ currents, determined from whole-cell patch clamp recordings, as the readout^24^. The system had two main advantages. First, we have extensively characterized both heterologous and native expression of FFAR3/FFAR2 in rat sympathetic neurons^12^. Second, activation of endogenous FFAR3 in the sympathetic nervous system is potentially important for a variety of physiological functions^12^,^25^.

An exemplar of the time course and current traces from the assay is illustrated in Fig. 6B,C, respectively, for an SCG neuron expressing the reference FFAR3 (GPR41.1) ORF. Ca^2+^ currents, arising primarily from N-type (Ca_V_2.2) channels^26^, were evoked with the voltage protocol illustrated in Fig. 6C (bottom). The percent inhibition of the current in the first test pulse (Fig. 6B,C; filled circle) in the presence of agonist (C3) provides the data for the concentration response curves (Fig. 6D) and maximal efficacy plot (Fig. 6E). The time course reveals the rapid onset and recovery of response characteristic of lower affinity GPCRs in this system^27^,^28^. The similarity of response to the first and last maximal concentration application indicate minimal tachyphylaxis to propionate. Also plotted in Fig. 6B (lower), the facilitation ratio or postpulse/prepulse amplitude ratio shows an increase during propionate application. This form of voltage-dependent inhibition is characteristic of Ca^2+^ channel modulation mediated by Gβγ^24^,^29^,^30^,^31^.

The concentration-response curves (Fig. 6D) illustrate the mean prepulse Ca^2+^ current inhibition for the labeled haplotypes versus log [C3 μM]. Expression of the reference FFAR3 sequence (41.1; purple filled circles) and the most abundant GPR42 sequence (42.1.1; S346N; red filled squares) produced similar results. The mean (95% CI range) EC_50_s for C3 were 11.1 (8.6–14.3) and 14.7 (10.5–20.4) μM, respectively. Conversely, haplotypes 42.2 (red filled triangle) and 42.3 (red inverted triangle; the current GPR42 reference sequence) exhibited decreased potency with EC_50_s for C3 of 65.9 (38.2–114) and 73.4 (53.8–100) μM, respectively. The maximal efficacy for Ca^2+^ current inhibition, determined during 1 mM C3 application, is illustrated in Fig. 6E. Application of 1 mM C3 to control neurons (i.e., not injected with cDNA) produced a small mean inhibition (8.3 ± 1.1%; n = 12) consistent with previous results^12^ suggesting a low endogenous expression of FFAR3. Similar maximal Ca^2+^ current inhibition was seen when expressing 41.1 (66.7 ± 2.7; n = 10), 42.1 (69.1 ± 2.9; n = 10), 42.2 (64.4 ± 4.1; n = 10), and 42.3 (69.6 ± 2.5; n = 13). Note that both haplotypes 42.2 and 42.3 contain the R174W substitution that was previously shown to result in a null phenotype^2^. A fourth GPR42 haplotype, 42.9 (red filled diamonds), produced a small inhibition (9.1 ± 1.3%; n = 10) that was not significantly different from the control.

Taken together with the evidence for transcription of GPR42 (Fig. 5), these results suggest that at least half of the GPR42 haplotypes sequenced (42.1.1–42.1.3) would result in a functional protein. Moreover, haplotypes containing the R174W, including the current GPR42 reference sequence (42.3), were shown to have decreased potency when compared with FFAR3 (41.1) but equivalent efficacy. Thus, the great majority of GPR42 haplotypes would be predicted to be functional.

## Discussion

Although the impact of FFAR3 and GPR42 on human physiology remains to be clarified, there is sufficiently promising rodent data to promote development of pharmacological agents that act on FFAR3^32^,^33^,^34^. For most Group A GPCRs, the ORF variant landscape is well defined due to efforts such as the 1000 Genomes initiative^35^. For example, FFAR2, from 1000 Genomes data, has two non-synonymous variants with a global minor allele frequency (MAF) of 1% or greater (dbSNP: rs409093, 4.8%; rs73931123, 1%) while FFAR1 has one (dbSNP: rs2301151, 12%). These frequencies are in general agreement with the “one SNP (>1%) per 1000 bp of sequence” estimate of global human sequence variation^36^. As described earlier, the tandem segmental duplication of FFAR3 in primates and the persistence of high sequence identity with the duplicated GPR42 ORF confounds short-read NGS sequencing efforts. Hence, only the reference sequence for FFAR3 (Genbank: NP_005295) is examined in most *in vitro* studies. Our data expose a complex genetic picture with high variability in both FFAR3 and GPR42 ORFs, copy number polymorphism in GPR42 resulting from a frequent structural variation, and evidence for reclassifying GPR42 from a pseudogene to a functional gene.

### Evidence supporting GPR42 as a functional gene

GPR42 was originally classified as a pseudogene based on non-functionality when the reference ORF was expressed in model systems^2^. A subsequent study^18^, however, revealed that the majority (61%) of GPR42 alleles examined did not possess the R174W polymorphism implicated in the inactive phenotype and thus questioned the pseudogene characterization. Our data indicate that GPR42 should be re-categorized as a functional gene based on the following observations. First, we found that only 31.9% of GPR42 alleles sequenced contained the R174W polymorphism. Second, the most common GPR42 haplotype (50.6%) differed from FFAR3 by just a single residue at the extreme C-terminus (S346N). Third, processed mRNA coding for GPR42 was found in human sympathetic ganglia and colon. Fourth, a common polymorphism (A919G; 30.8%) in GPR42 that was absent from FFAR3 was seen in colon RNA-seq databases (Illumina Body Map 2.0). Fifth, the most common GPR42 variant (42.1.1) was functionally equivalent to FFAR3 (41.1) in terms of potency and efficacy when assayed for Ca^2+^ channel modulation in rat sympathetic neurons. Sixth, GPR42 variants containing the R174W polymorphism, including the current GPR42 reference sequence, displayed less potency (for C3) but equal efficacy in the sympathetic neuron Ca^2+^ channel assay. This is in contrast to previous work^2^ suggesting complete inactivity of the GPR42 reference sequence. The reasons for the discrepancy are unclear, however, the assays used were very different (e.g., yeast reporter, GTP-γ-S binding) which might account for the different outcomes.

Definitive evidence for GPR42 function requires detection of the protein product in tissue with functional response to agonist. However, given the high degree of sequence identity, discrimination from FFAR3 will be challenging. Knockout of GPR42 or knock-in of a reporter construct might also provide important evidence. Unfortunately, GPR42 is only found in primates and thus common genetically alterable mammals (e.g., mice) are unsuitable. Newer genetic methodologies such as CRISPR/Cas9 allow genetic alteration in primates^37^, hence such experiments are feasible. In that regard, a sequence corresponding to an apparent GPR42 ORF is present in the rhesus macaque genome (Assembly BGI CR_1.0/rehMac3; Chr19: 41973858–41974878) that contains a single base deletion at nucleotide position 97. The predicted frame shift resulting from the deletion would likely inactivate the protein. Although the rhesus genome has limitations^38^, we have confirmed the presence of this deletion in five rhesus gDNA samples (of both Chinese and Indian origin; unpublished observation). Hence, based on current information, rhesus GPR42 is a genuine nonfunctional pseudogene.

### Copy number polymorphism in GPR42

The initial description of the human GPR40–43 family^17^ included sequencing of a cosmid clone, U62631, that appeared to be missing a region containing the GPR42 ORF. Another clone, AC002511, contained GPR42 and the authors proposed that this region might represent a polymorphic insert. Later, the reference human genome was shown to contain GPR42 and this anomaly received little further attention. In fact, the U62631 entry was edited to end within the GPR42 3′-UTR (Genbank: U62631.2). Ironically, we found evidence supporting a 12.5 kb deletion that conformed with the original U62631 sequence. The deletion likely occurred via NAHR within the FFAR3/GPR42 duplicated region — a common mechanism in tandem segmentally duplicated genes^39^,^40^,^41^. The deletion was quite common in our cohort accounting for 18.8% of the potential GPR42 alleles. A single sample, B02, was deleted in both GPR42 alleles and thus represented a GPR42 knockout.

We found confirmatory evidence, a 12 kb deletion structural variant (dbVar: esv2678346), in 1000 genomes data. The deletion has a global frequency of 18.5% but showed large variation in cohorts from different ancestry (Fig. S3). Of interest, the deletion is absent from the Yoruba and Luhya populations (from Nigeria and Kenya, respectively) but well represented in cohorts of European ancestry. At present, there are no clinical assertions documented for this variant. The precise breakpoint(s) of the deletion is unclear because of the high sequence identity of the boundary regions. However the presence of FFAR3 haplotypes (Group 3) containing the S346N variation suggest that the deletion may have occurred within homologous regions of the FFAR3/GPR42 ORF thereby resulting in a chimeric FFAR3 with the C-terminus of GPR42. A recent study^42^ indicates that the GPR42 copy number may exceed two suggesting additional complexity within this region.

### Variation in FFAR3 and GPR42

The magnitude of sequence variation within FFAR3 was unexpected. The reference sequence (41.1; Genbank: NM_005304) accounted for only 30.4% of the alleles examined. The next 3 most frequent variants (41.2–41.4), accounting for an additional 52.7% of alleles, each contained a single residue alteration of unknown consequence. Variant 41.4 contains the D158N substitution that may produce constitutive activity^19^ while 41.8 contains the R174W substitution that appears to alter efficacy^2^ or potency (this study).

The number of variants identified for GPR42 was equally unexpected. The major sets of variants, referred to as S346N and Group 1, complicate the designation for GPR42. The most common variants (42.1.1–42.1.2) differ by only a single residue and our electrophysiological studies (Fig. 6) indicate similar function to FFAR3 (41.1). Conversely, the Group 1 variants are complex, the majority possessing 6–7 non-synonymous polymorphisms. There are variable sites common to both FFAR3 and GPR42 suggesting some form of gene conversion. Hence, the GPR42 polymorphisms may best be classified as a multisite variation rather than paralogous sequence variants^43^. The absolute conservation of the S346N variation in every GPR42 allele sequenced is a mystery. An orthologous variation does not appear in other primate FFAR3/GPR42 orthologs (unpublished observation) although appears to be present in Neanderthal^44^ and Denisovan^45^ genomes. The extreme C-terminal residue of Group A GPCRs does not usually dictate important functions except when part of a PDZ ligand^46^. FFAR3/GPR42, however, do not contain a canonical PDZ ligand C-terminal sequence. The finding that GPR42 variants containing the R174W polymorphism are functional was surprising and bolsters the case for abandonment of the pseudogene categorization. Although constructs containing R174W were approximately 6-fold less potent for C3, the abundance of SCFA in the lumen of the colon^47^ would suggest that sufficient endogenous ligand exists to activate these variants.

## Conclusion

FFAR3 has been implicated in energy metabolism, sympathetic function, diabetes, immune function, and interactions with the gut microbiota. A primate-specific segmental duplication produced a duplicon termed GPR42 which has received little attention after being classified as a pseudogene. Here, we present multiple lines of evidence indicating that the function of GPR42 needs to be consider in human studies. GPR42 is a complex multisite variant and is affected by a common deletion that results in copy number polymorphism. Together with the variation in FFAR3 uncovered here, there may be complex haplotypes and copy number variations, possibly specific to human subpopulations, that impact human health and well-being.

## Methods

### Molecular Biology

Human genomic DNA samples (B01-50) were a generous gift from David Goldman MD, NIH/NIAAA/LNG. The cohort was 87% Caucasian, 52% male, and physically healthy at the time of collection. Each participant had a physical examination and routine blood tests at the NIH Clinical Center which were all normal. Participants gave informed consent according to a human research protocol approved by the Human Research Committee of the National Institute on Alcohol Abuse and Alcoholism, NIH. Human genomic DNA from a Finnish population in the 1000 Genomes Collection was obtained from the Coriell Institute for Medical Research (MGP00001; https://www.coriell.org). Human celiac ganglia and colon tissue samples were obtained from National Disease Research Interchange (NDRI, http://ndriresource.org). All samples were de-identified. The NDRI samples were determined “Not Human Subjects Research” by the NIH Office of Human Subjects Research Protections (OHSRP #19912).

Oligonucleotide primers were purchased from IDT. All subsequent primer positions with respect to both FFAR3 and GPR42 genomic sequences are illustrated in Fig. S4. Primer sets to differentially amplify human FFAR3 or GPR42 from genomic DNA were based on previously published sequences^18^:

FFAR3 5′: 5′-CCCCAGAGATAATCCTGCACCAGT-3′;

GPR42 5′: 5′-TACAGGTACTATGACAATCCCTGTTCTG-3′;

ORF 3′: 5′-GAGGAAGCGCCCTGAGGATGAC-3′.

FFAR3 or GPR42 genomic fragments were amplified from 10–15 ng of genomic DNA template in a 25 μL reaction using Q5 polymerase Master mix (NEB). Reactions were assembled in 0.2 mL PCR tubes using an Eppendorf epMotion M5073 liquid handling robot. Cycling parameters were as follows: 1 hold at 98 °C for 30 s followed by 33 cycles at 98°C for 10 s, 62 °C for 10 s and 72 °C for 90 s, followed by a final extension at 72 °C for 120 s.

PCR products were either purified using the Qiafilter PCR Cleanup kit from Qiagen and sequenced (MacrogenUSA) or subcloned using the TOPO PCR blunt cloning kit (Life Technologies) and individual clones were sequenced. PCR fragments and individual subclones were sequenced using the following internal overlapping primers:

FFAR3/GPR42mFor: 5′-GTGGAGGCAGCCAATGGCATGC-3′

FFAR3/GPR42mRev: 5′-CAGTGGGTGGGCCACACTCAGG-3′.

Sequence data was analyzed using Sequencher and MacVector.

Paired genotype and transcript data were obtained as follows: Celiac ganglia were obtained frozen in PBS. The colon sample was provided in Optimal Cutting Temperature (OCT) compound. Samples were thawed in Hank’s balanced salt solution (pH 7.4), trimmed to remove extraneous material, cut into small (~100 mg) pieces and placed in 1 mL of RNALater solution (Qiagen) in 1.5 mL microcentrifuge tubes. Stabilized tissue samples were placed at 4 °C overnight and then stored at −20 °C until use. Prior to use, the 100 mg piece was removed from the RNALater and cut into two equivalent pieces. One piece was used for genomic DNA preparation with the DNeasy Blood & Tissue Kit (Qiagen) and the second for total RNA preparation using the RNeasy miniprep kit (Qiagen). For RNA, the tissue was homogenized by grinding with a glass rod directly in lysis buffer (RTL). Following total RNA isolation, RNA quality was assessed using the Agilent Bioanalyzer and cDNA was prepared using the QuantiTect Reverse Transcription Kit (Qiagen).

Amplification of transcripts from cDNA was performed with Q5 polymerase and the following primers:

FFAR3/GPR42ex1For: 5′-GCATTTGGGGTCTCAAAGAAGC-3′

ORF 3′: 5′-GAGGAAGCGCCCTGAGGATGAC-3′.

Primers were designed to flank the 200 bp intron located in the 5′-UTR as a means of discriminating properly spliced transcripts from any contaminating genomic DNA. Cycling parameters were as follows: 1 hold at 98 °C for 30 s followed by 33 cycles of 98 °C for 10 s, 62 °C for 10 s and 72 °C for 60 s, and a single hold at 72 °C for 120 s. PCR products were subcloned and sequenced as described above.

The following primers were used as controls for RNA/cDNA quality (actin) and neuronal identity of input cells (TH):

Human β-actin control primer set: 981bp amplicon

hACTex1f: 5′-CGACAACGGCTCCGGCATGTGC-3′

hACTex5r: 5′-GTACTTGCGCTCAGGAGGAGC-3′

Human Tyrosine Hydroxylase control primer set: 859bp amplicon

hTHex6f: 5′-GCAGGAAGCTGATTGCTGAG-3′

hTHex14r: 5′- GTGTCCAGCTCATCCTGGAC-3′

Copy Number Variation assays were performed on a Step One Plus real time PCR instrument (ABI) in quadruplicate as per manufacturers directions (ABI-Life Technologies) with 20 ng of genomic DNA. TaqMan® copy number qPCR assay #Hs04030486_cn was chosen from inventoried assays based upon location within the putative deleted region. Results were analyzed with ABI CopyCaller™ software.

PCR validation of the absence of the putative deleted genomic region was detected using primers FFAR3 5′ described above and a primer unique to the genomic region downstream from hGPR42. (R3: 5′-GTGATTCTCTTGCCTCGGCATC-3′). This primer set generated an approximately 3 kb fragment if a deletion was detected. PCR reaction conditions (Q5 polymerase- NEB) were: 1 hold at 98 °C for 30 s followed by 33 cycles of 98 °C for 10 s, 62 °C for 10 s and 72 °C for 90 s followed by a final extension at 72 °C for 120 s.

### Superior cervical ganglion (SCG) neuron dissociation and nuclear microinjection

All animal studies were conducted in accordance to the National Institutes of Health’s *Guidelines for Animal Care and Use*. SCG neurons from adult (6–12 week old) male Wistar rats were dissected and dissociated as described previously^48^. Briefly, animals were anesthetized by CO_2_ inhalation and decapitated before dissection. Two SCG per rat were removed, desheathed, cut into small pieces, and incubated in modified Earles’ balanced salt solution (EBSS) containing 1.7 mg/mL collagenase (CLS4; Worthington Biochemical), 0.6 mg/mL trypsin (Worthington Biochemical) and 0.1 mg/mL DNase I at 36 °C for 1 hour in a water bath shaker rotating at 110 rpm. The EBSS was supplemented with 3.6 g/L d-glucose and 10 mM HEPES. After incubation, neurons were mechanically dissociated by vigorously shaking the flask for 10 s. Neurons were centrifuged at 570 rpm for 6 min and resuspended in Minimum Essential Medium (MEM) supplemented with 10% fetal bovine serum and 1% penicillin-streptomycin (Life Technologies, Grand Island, NY) twice. Neurons were plated on poly-l-lysine coated tissue culture dishes and maintained in a humidified 95% air/5% CO_2_ incubator at 37 °C.

Three to six hours after dissociation, plasmid cDNA constructs were injected directly into the nucleus of SCG neurons as described previously^48^,^49^. Briefly, cDNA was injected with an Eppendorf FemtoJet microinjector and 5171 micromanipulator (Eppendorf, Hauppauge, NY) using an injection pressure and duration of 140–160 hPa and 0.3 s, respectively. Mammalian expression vectors encoding variants of FFAR3 or GPR42 were injected at a concentration of 10–100 ng/μL along with 5 ng/μL EGFP cDNA (Clontech, Mountain View, CA) to identify successfully injected neurons. Following injections, neurons were incubated overnight at 37 °C and electrophysiological experiments were performed the following day.

### Electrophysiology

Ca^2+^ -channel currents were recorded using a patch-clamp amplifier (Axopatch 200B, Molecular Devices, Sunnyvale, CA) and conventional whole-cell patch-clamp techniques^50^. Patch electrodes were pulled from borosilicate glass capillaries (1.65 mm OD, 1.20 mm ID, King Precision Glass, Claremont, CA) using a Model P-97 micropipette puller (Sutter Instrument, Novato, CA). The patch electrodes were coated with silicone elastomer (Sylgard 184, Dow Corning, Midland, MI) and fire-polished. A Ag/AgCl pellet connected to the bath solution via a 0.15 M NaCl/agar bridge was used as a ground. Voltage protocol generation and data acquisition were performed using a custom written program using Igor Pro software (version 6, WaveMetrics, Portland, OR). Cell membrane capacitance was cancelled and series resistance was compensated (>85% prediction and correction; lag set to 5 μs). Current traces were filtered at 2 kHz (−3 dB; 4-pole Bessel), digitized at 10 kHz with a 16-bit analog-to-digital converter board (ITC-18, HEKA, Bellmore, NY) and stored on the computer for later analyses. To measure modulation of Ca^2+^ -channels following G protein activation, a double-pulse protocol consisting of two 25 ms test pulses to +10 mV separated by a 50 ms conditioning pulse to +80 mV^51^ was evoked every 10 s from a holding potential of −80 mV.

For recording Ca^2+^ currents, patch pipettes were filled with an internal solution containing (in mM) 120 N-methyl-d-glucamine, 20 tetraethylammonium hydroxide (TEA-OH), 11 EGTA, 10 HEPES, 10 sucrose, 1 CaCl_2_, 14 Tris-creatine phosphate, 4 MgATP and 0.3 Na_2_GTP, pH 7.2 with methanesulfonic acid. External Ca^2+^ current recording solution consisted of (in mM) 140 methanesulfonic acid, 145 TEA-OH, 10 HEPES, 10 glucose, 10 CaCl_2_ and 0.0003 tetrodotoxin (TTX), pH 7.4 with TEA-OH.

A range of propionate (C3) solution concentrations was applied directly onto neurons using a custom-made gravity-fed perfusion system with separate perfusion lines feeding into a custom designed 7-bore glass capillary tube (manufactured by VitroCom, Mountain Lakes, NJ) connected to a fused silica capillary tube. A constant flow of external solution was applied onto cells during baseline recordings and switched to a C3 solution during test applications to avoid flow-induced artifacts. All C3 solutions were diluted to final concentrations from a 1 M stock solution on the day of experiment and the pH was checked (pH 7.4–7.45). All recordings were performed at room temperature (20–24 °C).

### Data analysis and statistical testing

Igor Pro software was used to analyze current traces and statistical tests were performed with GraphPad Prism 6 for Mac OS X (GraphPad Software, La Jolla, CA). Ca^2+^ current amplitude was measured isochronally 10 ms after the initiation of a test pulse to +10 mV. The facilitation ratio (FR) was determined as the ratio of postpulse to prepulse Ca^2+^ current. Individual data points or the mean ± SEM were represented on graphs. Concentration-response curves were fit using non-linear regression (GraphPad Prism 6) to a 2-parameter equation:


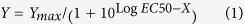


where *Y* is the agonist induced Ca^2+^ current inhibition, *Y*_*max*_ is the maximal inhibition, EC_50_ is the agonist concentration at half-maximal response, and *X* is log[agonist]. Statistical analysis between three or more groups was performed using a one-way analysis of variance (ANOVA) test followed by a Bonferroni’s multiple comparisons test. *P*-values < 0.05 was considered statistically significant.

## Additional Information

**How to cite this article**: Puhl, H. L. *et al.* Human GPR42 is a transcribed multisite variant that exhibits copy number polymorphism and is functional when heterologously expressed. *Sci. Rep.* **5**, 12880; doi: 10.1038/srep12880 (2015).

## Supplementary Material

Supplementary Information
