# Supplementary material for: Human GPR42 is a transcribed multisite variant that exhibits copy number polymorphism and is functional when heterologously expressed
Source: Sci Rep. 2015 Aug 11;5:12880. doi: 10.1038/srep12880 (PMC4531286; doi:10.1038/srep12880)
Supplement: Supplementary Information [file srep12880-s1.pdf]

Human GPR42 is a transcribed multisite variant that exhibits copy number polymorphism and is functional when heterologously expressed.

Abbreviated Title: Human *GPR42* is not a pseudogene

Henry L. Puhl III, Yu-Jin Won[†], Van B. Lu, and Stephen R. Ikeda[*]

Section on Transmitter Signaling, Laboratory of Molecular Physiology, National Institute on Alcohol Abuse and Alcoholism, National Institutes of Health, Bethesda, Maryland 20892-9411, USA

[†]Current address: Department of Life Science & Basic Science Institute for Cell Damage Control, Sogang University, Seoul, Republic of Korea C.P. 010-2703-8863

Supplemental Figures Legends

Figure S1. SNP positions for FFAR3/GPR42. (A) Table of SNP positions used for raster graphs shown in Figure 2, 4, and S2. Synonymous variants are highlighted in green. All variants detected were bi-allelic (ntΔ column). (B) GPCR snake plot (http://tools.gpcr.org) for human FFAR3 (41.1). Polymorphisms are coded as synonymous (green) or non-synonymous (red).

Figure S2. Unsorted sequence raster diagrams for FFAR and GPR42. (A and B) The diagrams were generated as in Figure 2 and 4 but alleles were not sorted. The sample order (left to right) was B01-50, CG01-05 (celiac-superior mesenteric ganglia), and C01 (colon). Each sample generated 2 alleles (or a deletion indicated by yellow fill in the column in B). As the sequencing was not phased, the order of alleles for an individual is arbitrary.

Figure S3. Distribution of the structural variant dbVar: esv2678346 from 1000 genomes data. Donut plots generated from 1000 genomes data (http://www.1000genomes.org). Blue represents no deletion, red heterozygous for deletion, and green homozygous for the deletion. The data were downloaded from the USCS genome browser [1] and individuals matched from ID numbers to specific cohort using a custom program. The number in the center of the donut represents the number of individuals sequenced.

Figure S4. Aligned genomic segments containing FFAR3 and GPR42. Positions 1 of FFAR3 and GPR42 correspond to position 35,848,787 and 35,861,281 of human chromosome 19 respectively (+ strand). Chromosome positions were determined using BLAT [2] with the Feb. 2009 build GRCh37 (UCSC version hg19). Exon 1 and the noncoding 5' end of exon 2 are highlighted (cyan). Start (green) and stop (red) codons are indicated. Nucleotide differences within the reference open reading frame of FFAR3 and GPR42 are highlighted in yellow. All FFAR3 and GPR42 primer locations used in the "Materials and Methods" section are labeled. Primers highlighted in green are from Liaw and Connolly [3], with numerical values indicating their prior designation.

1. Kent, W. J. *et al.* The human genome browser at UCSC. *Genome Res.* **12,** 996–1006 (2002).
2. Kent, W. J. BLAT--the BLAST-like alignment tool. *Genome Res.* **12,** 656–664 (2002).
3. Liaw, C. W. & Connolly, D. T. Sequence polymorphisms provide a common consensus sequence for *GPR41* and *GPR42*. *DNA Cell Biol* **28,** 555–560 (2009).

Figure S1

A

index	nt pos	ntΔ	aa (codon)	aaΔ
0	21	G→C	7(3)	Q→H
1	91	C→G	31(1)	L→V
2	131	A→G	44(2)	Q→R
3	133	C→T	45(1)	R→C
4	134	G→A	45(2)	R→H
5	230	A→G	77(2)	N→S
6	360	A→C	120(3)	P→P
7	377	G→A	126(2)	R→Q
8	398	G→A	133(2)	G→D
9	472	G→A	158(1)	D→N
10	520	C→T	174(1)	R→W
11	616	C→T	206(1)	R→C
12	624	G→A	208(3)	V→V
13	679	T→G	227(1)	L→ V
14	684	G→A	228(3)	A→A
15	753	C→T	251(3)	C→C
16	767	C→T	256(2)	A→V
17	768	G→A	256(3)	A→A
18	880	T→C	294(1)	L→L
19	919	A→G	307(1)	M→V
20	965	G→T	322(2)	R→L
21	1011	T→G	337(3)	T→T
22	1037	G→A	346(2)	S→N

B

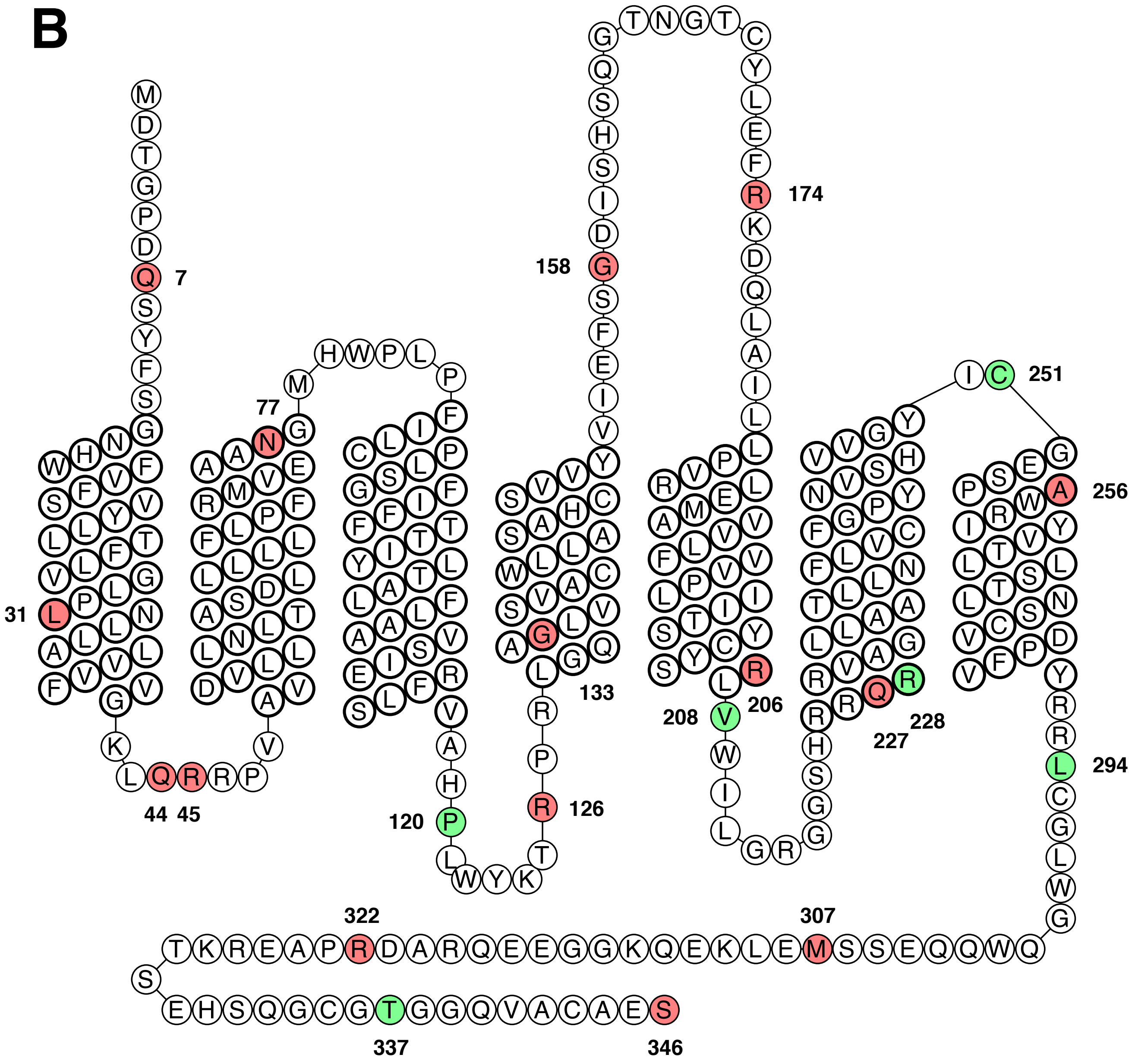

Figure S2

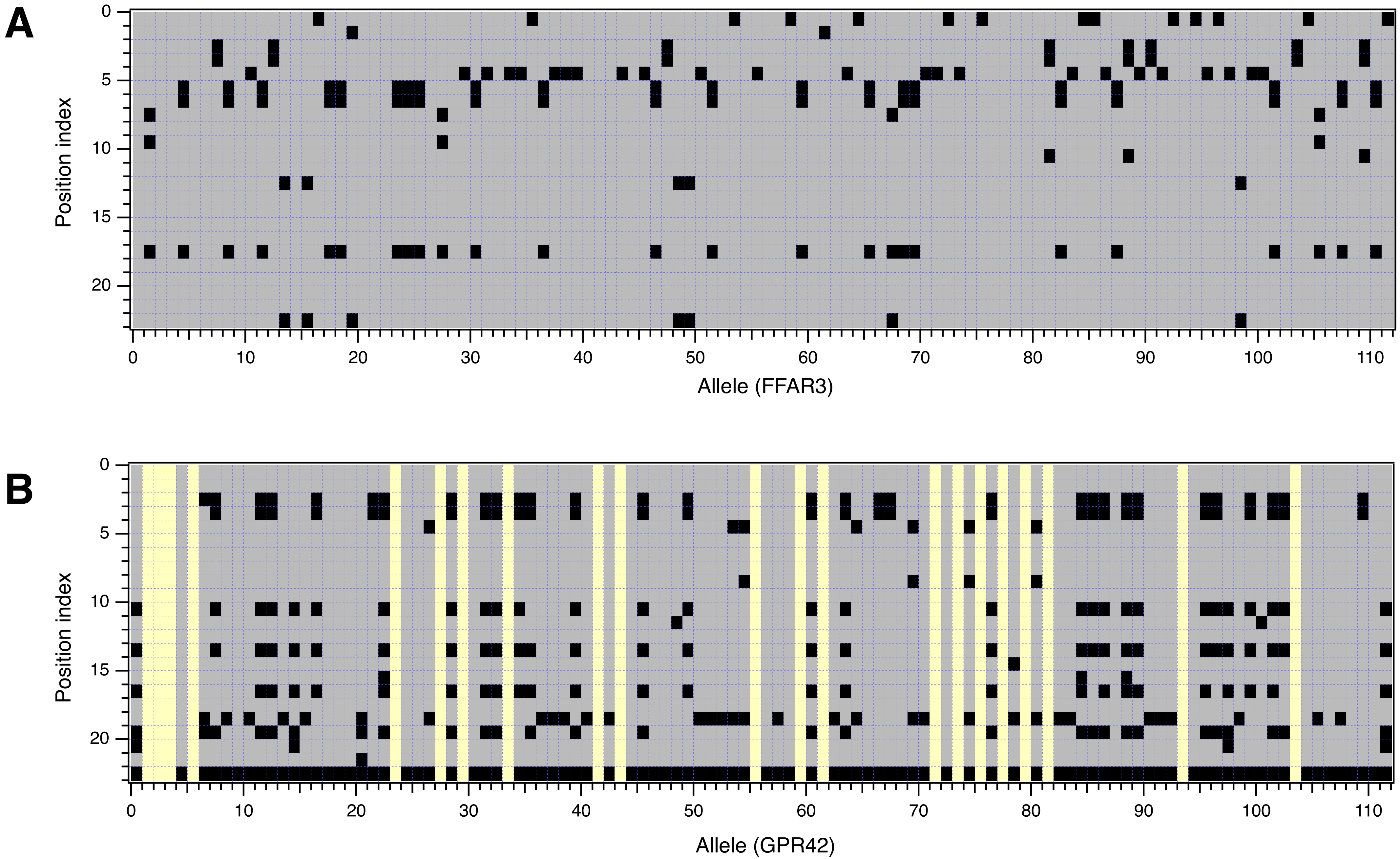
A
B
Position index
Allele (FFAR3)
Allele (GPR42)
0
5
10
15
20
0
10
20
30
40
50
60
70
80
90
100
110

Figure S3

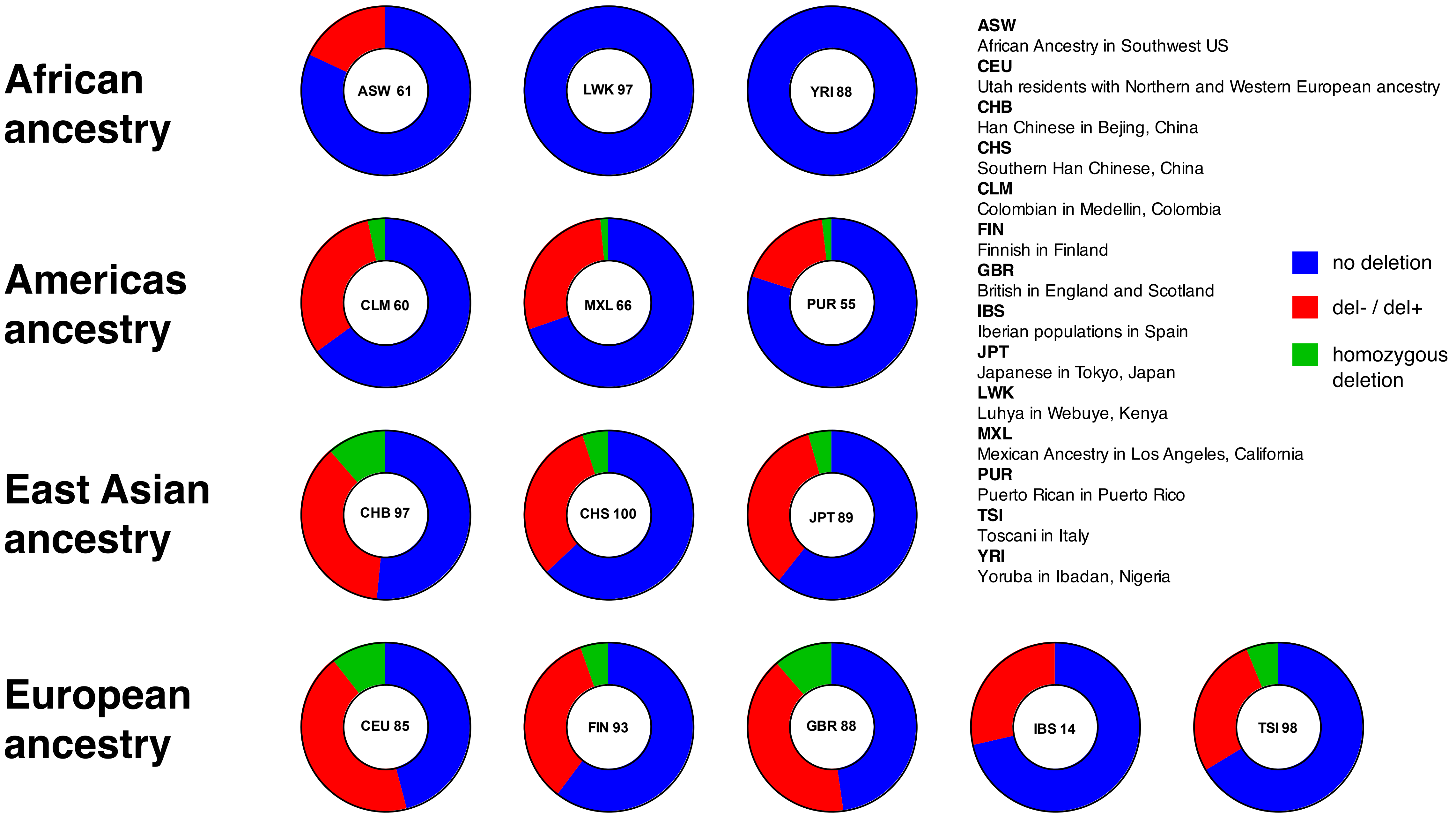
African ancestry
ASW 61
LWK 97
YRI 88
Americas ancestry
CLM 60
MXL 66
PUR 55
East Asian ancestry
CHB 97
CHS 100
JPT 89
European ancestry
CEU 85
FIN 93
GBR 88
IBS 14
TSI 98
ASW
African Ancestry in Southwest US
CEU
Utah residents with Northern and Western European ancestry
CHB
Han Chinese in Bejing, China
CHS
Southern Han Chinese, China
CLM
Colombian in Medellin, Colombia
FIN
Finnish in Finland
GBR
British in England and Scotland
IBS
Iberian populations in Spain
JPT
Japanese in Tokyo, Japan
LWK
Luhya in Webuye, Kenya
MXL
Mexican Ancestry in Los Angeles, California
PUR
Puerto Rican in Puerto Rico
TSI
Toscani in Italy
YRI
Yoruba in Ibadan, Nigeria
no deletion
del- / del+
homozygous deletion

Figure S4

```
FFAR3         1 TTATGACATAAAAGCGTTGCCCTAGAAGCAGTGTCTGCTATGGATTATGTGCCCATGAAA   60
GPR42         1 ATAGACAGTGGGGACTCCAAAGTGGGGG-AGGGAGGGCGGAGGACAAGG-----GTGGGA   54
                 **     *     *        * *  * ** *   **   ***  * *      **  *

FFAR3        61 AGGCAGCTGCTGTCATCATCGCCACCACCATTATTATTGTTATTATTATTACATTGTCAC  120
GPR42        55 AAACTGC--CTATTG-----GGTACTATGTTCACAATTTTGGTAATGGGTTCAATAGAAG  107
                *  * **  ** *        *  ** *    * *  *** *  * **   * ** *   *

                 FFAR3 5'(-887 to -864)
FFAR3       121 CCCAGAGAT-AATCCTGCACCAGTGACAGGGAAAATAGCAGCTGGCATCCGCTGCCTGCT  179
GPR42       108 CCCAAACCTCAGTATTATGCAATATACCCATGTAACA--AACCTGCACATG-----TGCT  160
                **** *  * * *  *   * *   **       ** *  * *  ***   *      ****

FFAR3       180 CACGACCACACGCCAGGC-TTCCGTCAAACCACTCAACATGTATTAGTAATCTTTTAATC  238
GPR42       161 CCCGAATCTAAAATAAATATTTTCAAAAAGCAATTGACCTATATTATTAACATTTTAATC  220
                * ***    *    *    **      *** ** *   ** * ***** ***  ********

FFAR3       239 GACCTACATAT-TGTTTTAATTTGCATGTGTTAATGCGTTGATCTATGAGATGGGTATTA  297
GPR42       221 TACCTGTATATATGTTTTAATCTGCATGTGTTCATGCTTTGAAATATGGTACAGGTACTA  280
                 ****  **** ********* ********** **** ****  ****  *  **** **

                GPR42 5'(-709 to -685)
FFAR3       298 TGATGAGCTCTGTTCTGCAGGGGAGAAAGCAGAAACATGGAGAATTTAAGTCATTTCCCC  357
GPR42       281 TGACAATCCCTGTTCTGTAGGGGAGGAAGAAGAGACACA-----------TCTCTTCCCC  329
                ***  * * ******** ******* *** *** ***              **  ******

FFAR3       358 CAAATCACAAAGTCAGGAAGAAACAGACCTCACGGAGCTCGCTCTCTGTCATTGCATCAC  417
GPR42       330 CACATCACAAAGTCAGGAAGGAACAGACCTCACGGAGTCCAGTC--TGTCACTGGCTCAT  387
                ** **************** ****************  *  **   ***** **  ***

FFAR3       418 ACTTCCTGCCCTTACAAGGCAAATTGGATAAATGCCATTCTAGAGAAGCAGACAAAATTC  477
GPR42       388 GCTTTGTGCCCTTAAAAGGCAAACTGGATAAGTGCCATTCTAGAGAATCAGACAAAATTC  447
                 ***  ******** ******** ******* *************** ************

FFAR3       478 AAGTGAAGAAGGGGAGAGG-----AAGACGTCGGCTGGGGCCTGCTTAGAGCATCCCAGC  532
GPR42       448 AAGTGGAAGAGAGGGGGAGGGGAAGAAGACGTCAGCTGGGGCCTGTTTGGAGCATCCCAGC  507
                ***** *  ** ** ****      ******** *********** ** ************

FFAR3       533 TGAGACTGCATGAGGAGGGAGGCACGCAGTTGTGGAATTTGTTCCCCTTTTAGCATGCTG  592
GPR42       508 TGAGGCTGCGCAAGGAGGGAGGCACGCAGTTGTGGAATTTGTTCCCCTTTTTGCATACTG  567
                **** ****   *************************************** **** ***

FFAR3       593 ACCAGCCCTGGCAACGGAGCTCAAGGCATCTATGTGCCACTGCTCAACAGTGAGTGACGT  652
GPR42       568 ACCAGCCCTGGCAACGGAGCTCAAGGCATCTTTGTGCCACTGCTCAACAGTGAGTGATGT  627
                ******************************* ************************* **

FFAR3       653 CATGGGCACGGCCAGGTCTTTATCAGTTCTGCCGGATAAATAGCCAACTGCACTAGGTCT  712
GPR42       628 CATGGGCACGGCCAGGTCTTTATCAGTTCTGCCGGATAAATAGCCAACTGCACTAGGTCT  687
                ************************************************************
                       Exon1                       FFAR3/GPR42ex1For
FFAR3       713 GGAGAGACAGCAAGGTGCTGTGCGACAGAGCATTTGGGGTCTCAAAGAAGCAGGTGAGCC  772
GPR42       688 GGAGAGACAGCAAGGTGCTGTGCGGCAGAGCATTTGGGGTCTCAAAGAAGCAGGTGAGCC  747
                ************************ ***********************************

FFAR3       773 TGGGCCCGAGGGGCTGGGTGGAGGAGCACCTTGGTGCTTCTCTGCTGGGGAAGGGACAGG  832
GPR42       748 TGGGCCCGAGGGGCTGGGTGGAGGAGCACCTTGGTGCTTCTCTGCTGGGGAAGGGACAGG  807
                ************************************************************

FFAR3       833 GGACAGGGCATGCTCAGGAAGACAGGCAGGCTGACCCCGCCTGGAAGGCACCCAGAGACA  892
GPR42       808 GGACAGGGCATGCTCAGGAAGACAGGCAGGCTGACCCCGCCTGGAAGGCACCCAGAGACA  867
                ************************************************************

FFAR3       893 AGAGGGGTGGGCGTAGTGACCTCGTGCCCTTTTAGGGGAGATGCTGCTGGCCAGAGGCCG  952
GPR42       868 AGAGGGGTGGGCGTAGTGACCTCGTGCCCTTTTAGGGGAGATGCTGCTGGCCAGAGGCCG  927
                ************************************************************
```

```
                                                             Exon2     Start
FFAR3      953 TTAGGGCCCCCACTACCAACTCCATGTTACTCTCTCTCACCAGTGGCCACCACCATGGAT 1012
GPR42      928 TTAGGGCCCCCACTACCAACTCCATGTTACTCTCTCTCACCAGTGGCCACCACCATGGAT  987
               ************************************************************

FFAR3     1013 ACAGGCCCCGACCAGTCCTACTTCTCCGGCAATCACTGGTTCGTCTTCTCGGTGTACCTT 1072
GPR42      988 ACAGGCCCCGACCAGTCCTACTTCTCCGGCAATCACTGGTTCGTCTTCTCGGTGTACCTT 1047
               ************************************************************

FFAR3     1073 CTCACTTTCCTGGTGGGGCTCCCCCTCAACCTGCTGGCCCTGGTGGTCTTCGTGGGCAAG 1132
GPR42     1048 CTCACTTTCCTGGTGGGGCTCCCCCTCAACCTGCTGGCCCTGGTGGTCTTCGTGGGCAAG 1107
               ************************************************************

FFAR3     1133 CTGCAGCGCCGCCCGGTGGCCGTGGACGTGCTCCTGCTCAACCTGACCGCCTCGGACCTG 1192
GPR42     1108 CTGCGGTGCCGCCCGGTGGCCGTGGACGTGCTCCTGCTCAACCTGACCGCCTCGGACCTG 1167
               **** * *****************************************************
                                                 FFAR3/GPR42mFor
FFAR3     1193 CTCCTGCTGCTGTTCCTGCCTTTCCGCATGGTGGAGGCAGCCAATGGCATGCACTGGCCC 1252
GPR42     1168 CTCCTGCTGCTGTTCCTGCCTTTCCGCATGGTGGAGGCAGCCAATGGCATGCACTGGCCC 1227
               ************************************************************

FFAR3     1253 CTGCCCTTCATCCTCTGCCCACTCTCTGGATTCATCTTCTTCACCACCATCTATCTCACC 1312
GPR42     1228 CTGCCCTTCATCCTCTGCCCACTCTCTGGATTCATCTTCTTCACCACCATCTATCTCACC 1287
               ************************************************************
                                                      FFAR3/GPR42mRev
FFAR3     1313 GCCCTCTTCCTGGCAGCTGTGAGCATTGAACGCTTCCTGAGTGTGGCCCACCCACTGTGG 1372
GPR42     1288 GCCCTCTTCCTGGCAGCTGTGAGCATTGAACGCTTCCTGAGTGTGGCCCACCCACTGTGG 1347
               ************************************************************

FFAR3     1373 TACAAGACCCGGCCGAGGCTGGGGCAGGCAGGTCTGGTGAGTGTGGCCTGCTGGCTGTTG 1432
GPR42     1348 TACAAGACCCGGCCGAGGCTGGGGCAGGCAGGTCTGGTGAGTGTGGCCTGCTGGCTGTTG 1407
               ************************************************************

FFAR3     1433 GCCTCTGCTCACTGCAGCGTGGTCTACGTCATAGAATTCTCAGGGGACATCTCCCACAGC 1492
GPR42     1408 GCCTCTGCTCACTGCAGCGTGGTCTACGTCATAGAATTCTCAGGGGACATCTCCCACAGC 1467
               ************************************************************

FFAR3     1493 CAGGGCACCAATGGGACCTGCTACCTGGAGTTCCGGAAGGACCAGCTAGCCATCCTCCTG 1552
GPR42     1468 CAGGGCACCAATGGGACCTGCTACCTGGAGTTCTGGAAGGACCAGCTAGCCATCCTCCTG 1527
               ******************************** ***************************

FFAR3     1553 CCCGTGCGGCTGGAGATGGCTGTGGTCCTCTTTGTGGTCCCGCTGATCATCACCAGCTAC 1612
GPR42     1528 CCCGTGCGGCTGGAGATGGCTGTGGTCCTCTTTGTGGTCCCGCTGATCATCACCAGCTAC 1587
               ************************************************************

FFAR3     1613 TGCTACAGCCGCCTGGTGTGGATCCTCGGCAGAGGGGGCAGCCACCGCCGGCAGAGGAGG 1672
GPR42     1588 TGCTACAGCCGCCTGGTGTGGATCCTCGGCAGAGGGGGCAGCCACCGCCGGCAGAGGAGG 1647
               ************************************************************

FFAR3     1673 GTGGCGGGGCTGTTGGCGGCCACGCTGCTCAACTTCCTTGTCTGCTTTGGGCCCTACAAC 1732
GPR42     1648 GTGGCGGGGCTGGTGGCGGCCACGCTGCTCAACTTCCTTGTCTGCTTTGGGCCCTACAAC 1707
               ************ ***********************************************

FFAR3     1733 GTGTCCCATGTCGTGGGCTATATCTGCGGTGAAAGCCCGGCGTGGAGGATCTACGTGACG 1792
GPR42     1708 GTGTCCCATGTCGTGGGCTATATCTGCGGTGAAAGCCCGGTGTGGAGGATCTACGTGACG 1767
               **************************************** *******************

FFAR3     1793 CTTCTCAGCACCCTGAACTCCTGTGTCGACCCCTTTGTCTACTACTTCTCCTCCTCCGGG 1852
GPR42     1768 CTTCTCAGCACCCTGAACTCCTGTGTCGACCCCTTTGTCTACTACTTCTCCTCCTCCGGG 1827
               ************************************************************

FFAR3     1853 TTCCAAGCCGACTTTCATGAGCTGCTGAGGAGGTTGTGTGGGCTCTGGGGCCAGTGGCAG 1912
GPR42     1828 TTCCAAGCCGACTTTCATGAGCTGCTGAGGAGGTTGTGTGGGCTCTGGGGCCAGTGGCAG 1887
               ************************************************************

FFAR3     1913 CAGGAGAGCAGCATGGAGCTGAAGGAGCAGAAGGGAGGGGAGGAGCAGAGAGCGGACCGA 1972
GPR42     1888 CAGGAGAGCAGCATGGAGCTGAAGGAGCAGAAGGGAGGGGAGGAGCAGAGAGCGGACCGA 1947
               ************************************************************

FFAR3     1973 CCAGCTGAAAGAAAGACCAGTGAACACTCACAGGGCTGTGGAACTGGTGGCCAGGTGGCC 2032
GPR42     1948 CCAGCTGAAAGAAAGACCAGTGAACACTCACAGGGCTGTGGAACTGGTGGCCAGGTGGCC 2007
               ************************************************************
```

```
                                  Stop                          ORF 3’(32 to 53)
FFAR3      2033 TGTGCTGAAAGCTAGGTCCTCCGGGGGAGGAGGGTGTAGCTGGCATGTCATCCTCAGGGC 2092
GPR42      2008 TGTGCTGAAAACTAGGTCCTCCGGGGGAGGAGGGTGTAGCTGGCGTGTCATCCTCAGGGC 2067
                ********** ********************************* ***************

FFAR3      2093 GCTTCCTCGCTCACGCCAGGAGGGACTTGGAGTGGCGAGCTGGGGCCCGATGGGGCTTGG 2152
GPR42      2068 GCTTCCTCGCTCACGCCAGGAGGGACTTGGAGTGGCGAGCTGGGGCCCGATGGGGCTTGG 2127
                ************************************************************

FFAR3      2153 GGGCAGAGTAGACATCTAGCCTCCCTAAGGGTATGCGCGCTAAAGCCCAGCTCTCGATCT 2212
GPR42      2128 GGGCAGAGTAGACATCTAGCCTCCCTAAGGGTATGCGCGCTAAAGCCCAGCTCTCGATCT 2187
                ************************************************************

FFAR3      2213 CACCTCCATCCCCATCCACCCACACACTATGGATTGGGCTCTGGGAAGGGGTCAGGGTGA 2272
GPR42      2188 CACCTCCATCCCCATCCACCCACACACTATGGATTGGGCTCTGGGAAGGGGTCAGGGTGA 2247
                ************************************************************

FFAR3      2273 GAGGCTGCTCTGGAGAACAATGAGGTCCTCATAGCAGCAGGCAGCTCCTGTGTTTTCTTG 2332
GPR42      2248 GAGGCTGCTCTGGAGAACAATGAGGTCCTCATAGCAGCAGGCAGCTCCTGTGTTTTCTTG 2307
                ************************************************************

FFAR3      2333 AGGGTGGCAGAGGAGCTAAGAGCAGTGCCCAGGGTCTGAGGGGGCTGCCCAGTGAGTGGC 2392
GPR42      2308 AGGGTGGCAGAGGAGCTAAGAGCAGTGCCCAGG-TCTGAGGGGGCTGCCCAGTGAGTGGC 2366
                ********************************* **************************

FFAR3      2393 AGGGGCAGGAGAGGGGAGAACCCCATCCTCAGAGCTGCTCCCAGCCAGCGAGTCAGGAGC 2452
GPR42      2367 AGGGGCAGGAGAGGGGAGAACCCCATCCTCAGAGCTGCTCCCAGCCAGCGAGTCAGGAGC 2426
                ************************************************************

FFAR3      2453 GGGGGAGACAGGGCTCCAGGGATGAGGCCGCATTCTGCTCCCACAGCGCCTTTTCCAGAA 2512
GPR42      2427 GGGGGAGACAGGGCTCCAGGGATGAGGCCGCATTCTGCTCCCACAGTGCCTTTTCCAGAA 2486
                ********************************************** *************

FFAR3      2513 AGTTCCCATTGCTCAATAAATGTGGATCATCAGAGACATTTATGAACAATGACAGAAGAA 2572
GPR42      2487 AGTTCCCATTGCTCAATAAATGTGGATCATCAGAGACATTTATGAACAATGACAGAAGAA 2546
                ************************************************************

FFAR3      2573 AAATTACCCAAATAAATGTGGAAGCAAGCAAAAGAGAACAGTGTTTCCTTCTTCTCCTGT 2632
GPR42      2547 AAATTACCCAAATAAATGTGGAAGCAAGCAAAAGAGAACAGTGTTTCCTTCTTCTCCTGT 2606
                ************************************************************

FFAR3      2633 TTTGTTCTGGTGGTGTGCTTGGGCCGGGTGGGACTGGTGGATGGAAGGAGAAAACACCAG 2692
GPR42      2607 TTTGTTCTGGTGGTGTGCTTGGGCCGGGTGGGACTGGTGGATGGAAGGAGAAAACACCAG 2666
                ************************************************************

FFAR3      2693 ACTCTGGAGGAAAAGGGCCAAACACCAGGATGCCTGGATGCTGGGAGAGGATCTGGCTTG 2752
GPR42      2667 ACTCTGGAGGAAAAGGGCCAAACACCAGGATGCCTGGATGCTGGGAGAGGATCTGGCTTG 2726
                ************************************************************

FFAR3      2753 CAGGGATGAAAATAACAGCTGCCCTGTCTAAAGGACTTGGCCTGACACATCATCTCCTTC 2812
GPR42      2727 CAGGGATGAAAATAACAGCTGCCCTGTCTAAAGGACTTGGCCTGACACATCATCTCCTTC 2786
                ************************************************************

FFAR3      2813 TATCTCTCAATAGCCCTGTGAGAGGTACCAGCATTATCCCCAGTTTCAGATGAAGGAGTG 2872
GPR42      2787 TATCTCTCAATAGCCCTGTGAGAGGTACCAGCATTATCCCCAGTTTCAGATGAAGGAGTG 2846
                ************************************************************

FFAR3      2873 GCCCAGAGAGGTGACACCTCCTACCTGAGATCCCATAGCTGGTGGGCGATTGAAGTGGGA 2932
GPR42      2847 GCCCAGAGAGGTGACATCTCCTACCCGAGATCCCATAGCTGGTGGGCGATTGAAGTGGGA 2906
                **************** ******** **********************************

FFAR3      2933 CCAGAAGCTGGTGTCAGTTGACTCTGACACCCATGCCCCTAAGCCACTCTGCTGTTCTCC 2992
GPR42      2907 CCAGAAGCTGGTGTCAGTTGACTCTGACACCCATGCCCCTAAGCCACTCTGCTGTTCTCC 2966
                ************************************************************

FFAR3      2993 ATCTGTGTGTCACCTGTGGTCACCTGGCCCAGTCAAATGTCCCAAGTCAGATAAGTCTGT 3052
GPR42      2967 ATCTGTGTGTCACCTGTGGTCACCTGGCCCAG--------------TCAGATGAGGCTGT 3012
                ********************************              ****** ** ****

FFAR3      3053 CTGAGCAGGACAAATATAAACAGGCCATGATAAGAAACAGAACTAAATGCAAAGCGTATT 3112
GPR42      3013 CTGAGCAGGACAAATGCAAACAGGCTGTGATAAGGAACAGAAGTAAATGCAAAGCACAGT 3072
                ***************  ********  ******* ******* ************  * *
```

```
FFAR3      3113 GACTATTTGTGTGGGTTCAACCTTTTCCCAGTAAAAAAGCCCTCTTGGAAAAAAAGAAAA 3172
GPR42      3073 GAGTATCTGTGTGAGTGCATCCTCTCCCCAGTAAAGAAGACTTCTTG-AAAAGTATAGAT 3131
                ** *** ****** ** ** *** * ********* *** * ***** ****  * * *

FFAR3      3173 AAAGATAATTAGTCAGCACAATGGACTTCAGGTTTCTGCAAAGTCAGAGA--GATGTTCC 3230
GPR42      3132 TACGATACTTGGAGTCAATGATTGGG--CAGGTGACT-CACAGTGGGCACCCGATGCTCC 3188
                 * **** ** *     *  ** *    *****  ** ** ***  *     **** ***

FFAR3      3231 TCAGAGCTCTATTCATCAAAATATGGCCATTCAGGAAGTTTTCATGTTCCCTGAGTGGTG 3290
GPR42      3189 TC-GATCTTAGCT------AATGCAGGATCTCTTCAACTTTTTACATCCTT------GTC 3235
                ** ** **    *      ***   *    **   ** **** *  * *        **

FFAR3      3291 GCCCATGAGAAATAGCTGGTCAGGACCTCCTCCCCAAGCAAGGCCACCTGAGACTCGCCA 3350
GPR42      3236 CCCTTCAGAAGCCTCTTTGGATATACATCTTTCCTACACAGCACATTCCTCTGCCTGCTT 3295
                 **      *      * *     ** ** * ** *  **   *   *     *  **

FFAR3      3351 GCCCCTGTTTGCATGCTGTCAGCAGTATTTCAGAAGCATTTGTTGAACACCTAT-TGCAT 3409
GPR42      3296 ATGCA-ATTTCAGTGCAACCAACA-CACTGTCGGTTTATGTGTTGTGGCCCTTTACAAAG 3353
                   *   ***   ***   ** **  * *   *    ** *****    *** *    *
                                                                      R3
FFAR3      3410 GCAGA---TGGCTGGGCTCAGAACTTCGGG-----GAGTTCGACACTAAGGTCTTTAAAT 3461
GPR42      3354 ACAGAAACTGGCTGGGCGCAGTGGCTCATGCCTATAATCCCAGCACTTTGGGATGCCGAG 3413
                 ****   ********* ***    **  *      *   *  ****  **  *    *

FFAR3      3462 ATCTGCTTCCTCCCTGGGGTTAAGTGTGCCGGGAGAATGAAGACTGGCTGTGTGTGGGGA 3521
GPR42      3414 GCAAGAGAATCACTTGAGGTCAGGAGTTT---GAGACC--AGCCTGGCCAACATGGTGAA 3468
                    *       * ** *** * * **     ****    ** *****       * * *

FFAR3      3522 AGTGGATTTCAGGTGGAAGGAGAGGGACTCTGTGGAGAGTTGAAACCATGAGCTGTCTCT 3581
GPR42      3469 ACTTTGTCTCTACTAAAAGTACAAATATTAGCCGCATA--TGGCAGCATGCACCT----- 3521
                * *   * **   *  *** * *   * *    * * *  **  * ****  *
```
